# The Incidence and Outcomes of Out-of-Hospital Cardiac Arrest During the COVID-19 Pandemic in South Korea: Multicenter Registry Study

**DOI:** 10.2196/52402

**Published:** 2024-06-24

**Authors:** Heekyung Lee, Jaehoon Oh, Hyuk Joong Choi, Hyungoo Shin, Yongil Cho, Juncheol Lee

**Affiliations:** 1 Department of Emergency Medicine Hanyang University College of Medicine Seoul Republic of Korea; 2 Department of Emergency Medicine Hanyang University Guri Hospital Gyeonggi-do Republic of Korea; 3 Department of Emergency Medicine Hanyang University Hospital Seoul Republic of Korea

**Keywords:** heart arrest, cardiopulmonary resuscitation, SARS-CoV-2, mortality, outpatient, cardiac arrest, multicenter registry study, out-of-hospital cardiac arrest, heart attack, observational study, adult, older adults, analysis, pandemic, prepandemic, endemic, defibrillator, COVID-19

## Abstract

**Background:**

The COVID-19 pandemic has profoundly affected out-of-hospital cardiac arrest (OHCA) and disrupted the chain of survival. Even after the end of the pandemic, the risk of new variants and surges persists. Analyzing the characteristics of OHCA during the pandemic is important to prepare for the next pandemic and to avoid repeated negative outcomes. However, previous studies have yielded somewhat varied results, depending on the health care system or the specific characteristics of social structures.

**Objective:**

We aimed to investigate and compare the incidence, outcomes, and characteristics of OHCA during the prepandemic and pandemic periods using data from a nationwide multicenter OHCA registry.

**Methods:**

We conducted a multicenter, retrospective, observational study using data from the Korean Cardiac Arrest Resuscitation Consortium (KoCARC) registry. This study included adult patients with OHCA in South Korea across 3 distinct 1-year periods: the prepandemic period (from January to December 2019), early phase pandemic period (from July 2020 to June 2021), and late phase pandemic period (from July 2021 to June 2022). We extracted and contrasted the characteristics of patients with OHCA, prehospital time factors, and outcomes for the patients across these 3 periods. The primary outcomes were survival to hospital admission and survival to hospital discharge. The secondary outcome was good neurological outcome.

**Results:**

From the 3 designated periods, a total of 9031 adult patients with OHCA were eligible for analysis (prepandemic: n=2728; early pandemic: n=2954; and late pandemic: n=3349). Witnessed arrest (*P*<.001) and arrest at home or residence (*P*=.001) were significantly more frequent during the pandemic period than during the prepandemic period, and automated external defibrillator use by bystanders was lower in the early phase of the pandemic than during other periods. As the pandemic advanced, the rates of the first monitored shockable rhythm (*P*=.10) and prehospital endotracheal intubation (*P*<.001) decreased significantly. Time from cardiac arrest cognition to emergency department arrival increased sequentially (prepandemic: 33 min; early pandemic: 35 min; and late pandemic: 36 min; *P*<.001). Both survival and neurological outcomes worsened as the pandemic progressed, with survival to discharge showing the largest statistical difference (prepandemic: 385/2728, 14.1%; early pandemic: 355/2954, 12%; and late pandemic: 392/3349, 11.7%; *P*=.01). Additionally, none of the outcomes differed significantly between the early and late phase pandemic periods (all *P*>.05).

**Conclusions:**

During the pandemic, especially amid community COVID-19 surges, the incidence of OHCA increased while survival rates and good neurological outcome at discharge decreased. Prehospital OHCA factors, which are directly related to OHCA prognosis, were adversely affected by the pandemic. Ongoing discussions are needed to maintain the chain of survival in the event of a new pandemic.

**Trial Registration:**

ClinicalTrials.gov NCT03222999; https://classic.clinicaltrials.gov/ct2/show/NCT03222999

## Introduction

At the end of December 2019, the outbreak of SARS-CoV-2 emerged in Wuhan, China, leading to the global pandemic of COVID-19 [1,2]. As of August 2023, more than 769 million confirmed cases and almost 7 million cumulative fatalities have been attributed to COVID-19 [3]. The emergence of SARS-CoV-2 variants and mutations has played a significant role in the pandemic’s persistence [4], and even after the end of the global pandemic, the risk of new variants and surges remains [5].

According to the current out-of-hospital cardiac arrest (OHCA) guidelines, the chain of survival (CoS) has been constantly emphasized to improve outcomes for patients with OHCA [6]. The OHCA CoS includes rapid recognition and activation of emergency response, early high-quality cardiopulmonary resuscitation (CPR) and defibrillation, and effective advanced life support interventions [6]. Each prehospital step experienced concurrent disruptions during the COVID-19 pandemic, resulting in a disruption of the OHCA CoS worldwide [7-9]. Notably, there was an increase in the incidence of OHCA, while rates of bystander CPR and automated external defibrillator (AED) use rate declined, especially during periods of heightened community transmission [10,11]. Additionally, a study using emergency medical services (EMS) data of South Korea reported that the proportion of prehospital return of spontaneous circulation (ROSC) decreased during the pandemic period [12].

During the pandemic era, outcomes of OHCA, including survival and neurological outcomes, deteriorated [9,10,13]. A previous meta-analysis reported that the ROSC rate, survival to hospital admission, and hospital discharge with good neurologic outcome (GNO) were significantly lower during the pandemic compared to the prepandemic period [10]. One recent registry-based cohort study reported that the survival rate was significantly decreased while the bystander CPR rate was stable [14]. Another study reported higher incidence of OHCA and decreased bystander CPR in regions with a high burden of pandemic [15]. A population-based nationwide study in Japan also reported that the pandemic is related to poorer neurologic outcomes and less AED use in patients with OHCA [16]. These poor outcomes could be closely related to changes in the prehospital phase during the pandemic, especially when considering the nature of OHCA.

Previous research has explored the impact of the COVID-19 pandemic on OHCA incidence, outcomes, and prehospital factors [10,17-19]. Analyzing the characteristics of OHCA during the pandemic is important to prepare for the next pandemic and to avoid repeated negative outcomes. However, previous studies have yielded somewhat varied results, depending on the health care system or the specific characteristics of social structures. Here, we aimed to investigate and compare the incidence, outcomes, and characteristics of OHCA between the prepandemic and pandemic periods, using data from a nationwide multicenter registry.

## Methods

### Study Design

We conducted a multicenter, retrospective, observational study using data from the Korean Cardiac Arrest Resuscitation Consortium (KoCARC) registry. This nationwide registry collates OHCA cases in South Korea in alignment with Utstein-style templates and involves a collaborative research network among multiple hospitals. The study included patients with OHCA who were transported to the emergency department (ED) by EMS and underwent resuscitation. Inclusion was limited to patients diagnosed with a medical cause identified by emergency physicians. Meanwhile, individuals with terminal illnesses, in hospice care, who are pregnant, or with a “Do Not Resuscitate” card were excluded. Patients with OHCA resulting from nonmedical causes, such as trauma, poisoning, burns, drowning, asphyxia, or hanging, were also excluded. Data were collected using a standardized registry form and uploaded to a web-based electronic database. The quality of the registry was monitored using a quality management committee to improve its reliability and integrity.

### Ethical Considerations

The project was registered at ClinicalTrials.gov with the identifier NCT03222999 and received ethical approval from the institutional review boards of the 62 participating hospitals. Given the nature of this study, the requirement for informed consent was waived by the institutional review boards. All personal information of patients with OHCA enrolled in the registry was anonymized. There was no direct compensation for the included patients.

### Study Population

This study included OHCA cases in South Korea registered in the KoCARC database in 3 distinct 1-year periods: the prepandemic period, early phase of the pandemic, and late phase of the pandemic. The KoCARC releases a year’s worth of data sets annually, with the period starting in July and ending in June of the following year. The prepandemic period was defined as the year leading up to the reporting of the first COVID-19 case (from January 1 to December 31, 2019). The early phase of the pandemic corresponds to the first data set released after the declaration of the pandemic (from July 1, 2020, to June 30, 2021). The late phase of the pandemic encompasses the most recent data set (from July 1, 2021, to June 30, 2022). Patients aged <18 years were excluded.

### Data Extraction and Definition

Previously published literature has provided comprehensive details regarding the KoCARC database, including information on data elements and quality assurance [20]. We extracted the following data from the KoCARC database: (1) patient characteristics: age, sex, witnessed arrest, arrest location at home or residence, bystander response (CPR and AED), first monitored shockable rhythm, and prehospital management (defibrillation, adrenaline use, and advanced airway [supraglottic airway or endotracheal intubation]); and (2) prehospital time factors: cardiac arrest recognition to ED arrival, EMS call to EMS arrival, arrest place arrival to departure, arrest place departure to ED arrival, and cardiac arrest recognition to prehospital ROSC.

### Outcome Variables

Primary outcomes were survival until hospital admission and survival to hospital discharge. We compared survival rates between the prepandemic period and the early and late pandemic periods and investigated the factors affecting survival rates. The secondary outcome was a good neurological outcome (GNO), which was defined as a Cerebral Performance Category (CPC) score of 1 or 2 at the time of hospital discharge. A CPC score of 1 denotes good cerebral performance, meaning patients are conscious, alert, and capable of functioning with only minor neurological or psychological deficits. A CPC score of 2 indicates moderate cerebral disability, with patients being conscious, possessing adequate cerebral function for independent daily activities, and having the ability to work in sheltered environments. A CPC score of 5 corresponds to mortality, specifically defined as death or brain death.

### Statistical Analysis

This study used data from the KoCARC registry, which were meticulously compiled and organized using a spreadsheet application (Excel 365; Microsoft). Continuous baseline variables were presented as median and IQR, and their normal distribution was assessed using the Shapiro-Wilk test. In instances where the data deviated from the normality assumptions, the Kruskal-Wallis test was used to compare the groups and the Mann-Whitney *U* test was used for a post hoc test. Categorical variables were expressed as absolute counts and percentages and analyzed using the chi-square test. A significance threshold of *P*<.05 was adopted to establish statistical significance.

To ascertain the independent association between variables and neurological and survival outcomes, multivariate analysis was performed using logistic regression. Furthermore, to validate the calibration of the logistic model, the Hosmer-Lemeshow test was applied. The statistical software packages SPSS (version 27.0 for Windows; IBM) and MedCalc (version 17.2; MedCalc Software) were used to perform all statistical computations.

## Results

**Figure 1:**
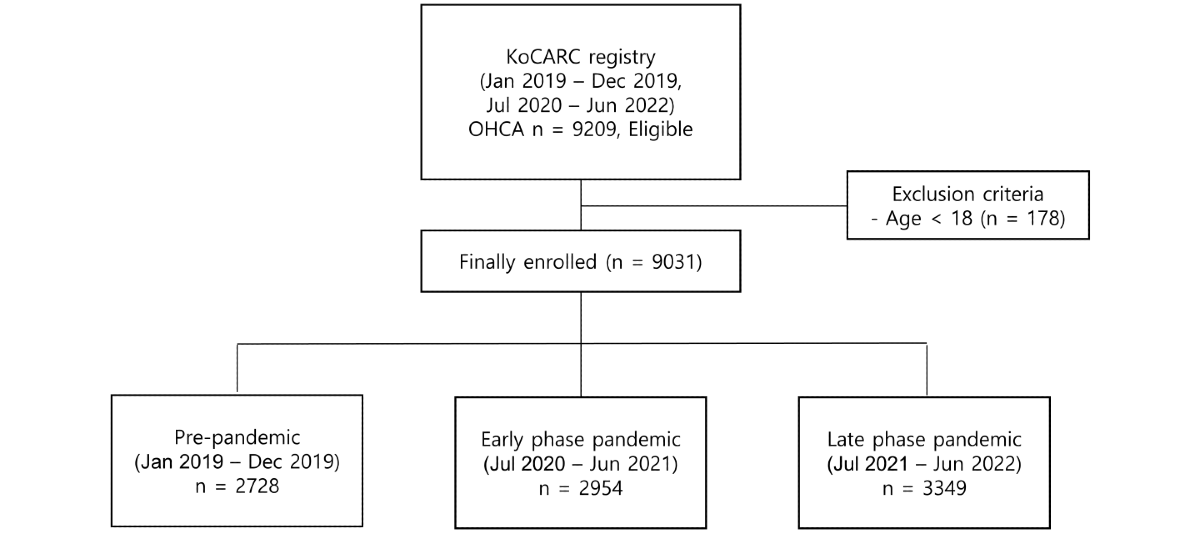
Flowchart of the study. KoCARC: Korean Cardiac Arrest Research Consortium; OHCA: out-of-hospital cardiac arrest.

**Figure 2:**
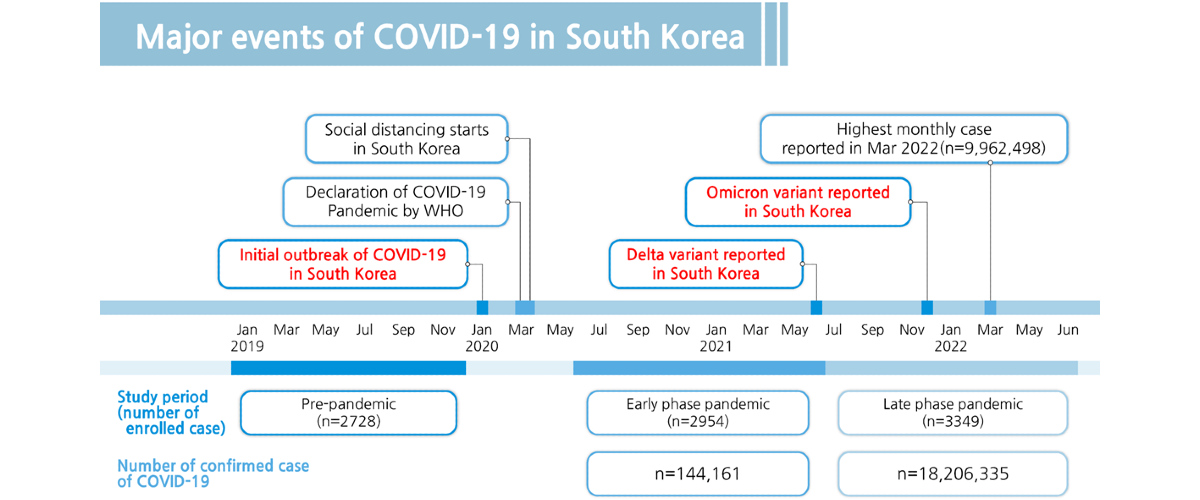
Major events and number of confirmed cases of COVID-19 in South Korea during the study periods (from January 2019 to June 2022). WHO: World Health Organization.

From the 3 designated periods, a total of 9209 patients with OHCA were registered. After excluding patients aged <18 years, 9031 patients with OHCA were deemed eligible. The number of patients included in each period was as follows: 2728 in the prepandemic period, 2954 in the early phase of the pandemic, and 3349 in the late phase of the pandemic (Figures 1 and 2). There was a sequential increase in the incidence of OHCA at the participating hospitals throughout the pandemic.

The baseline and prehospital OHCA characteristics from the 3 periods, along with intergroup comparisons, are summarized in Table 1. Age was significantly higher in the late phase pandemic period compared to the prepandemic or early phase pandemic periods (prepandemic: 72 y; early pandemic: 72 y; and late pandemic: 73 y; *P*=.02). However, there was no significant difference in the sex distribution across the periods (*P*=.55). Witnessed arrests (*P*<.001) and arrests at home or residence (*P*=.001) were significantly higher during the pandemic compared to the prepandemic period. Although bystander CPR was not statistically different among the 3 periods (*P*=.43), AED use by bystanders was lower in the early phase of the pandemic compared to the other periods. The rate of the first monitored shockable rhythm and prehospital defibrillation decreased sequentially throughout the study period. The prehospital endotracheal intubation rate significantly decreased as the pandemic progressed (prepandemic: 246/2728, 9%; early pandemic: 154/2954, 5.2%; and late pandemic: 116/3349, 3.5%; *P*<.001), resulting in a significant increase in supraglottic airway use (prepandemic: 1685/2728, 61.9%; early pandemic: 2158/2954, 73.3%; and late pandemic: 2408/3349, 72.6%; *P*<.001).

During the pandemic, pre–hospital phase time factors were extended compared to the prepandemic period. The time from cardiac arrest recognition to ED arrival increased sequentially (prepandemic: 33 min; early pandemic: 35 min; and late pandemic: 36 min; *P*<.001). Other time-related factors also worsened during the pandemic, including EMS call to arrival, arrest place arrival to departure, and arrest place departure to ED arrival.

Primary and secondary outcomes are summarized in Table 2. As the pandemic progressed, both survival and neurological outcomes worsened. The survival-to-discharge rate exhibited the most pronounced statistical variation (prepandemic: 385/2728, 14.1%; early pandemic: 355/2954, 12%; and late pandemic: 392/3349, 11.7%; *P*=.01). The survival-to–hospital admission rate was 24.3% (815/3349) in the late phase of the pandemic, which was significantly lower than that in the prepandemic period (*P*=.02). However, there was no statistically significant difference between the prepandemic and early phase pandemic periods (734/2728, 26.9% vs 734/2954, 24.8%; *P*=.08). Both the survival-to-discharge (*P*=.02 and *P*=.005, respectively) and GNO (both *P*=.03) rates were significantly lower in the early and late phase pandemic periods than in the prepandemic period. Additionally, none of the outcomes differed significantly between early and late phase pandemic periods (all *P*>.05).

**Table 1:** Baseline and prehospital characteristics of patients with out-of-hospital cardiac arrest enrolled in the nationwide multicenter registry of South Korea.

Variables		Prepandemic period^a^ (n=2728)	Early phase pandemic period^b^ (n=2954)	Late phase pandemic period^c^ (n=3349)	*P* value^d,e^ (pre- vs early pandemic)	*P* value ^d,e^ (pre- vs late pandemic)	*P* value^d,e^ (early vs late pandemic)	*P* value^e,f^
Age (y), median (IQR)^g^		72 (59-81)	72 (59-81)	73 (60-82)	.45	.06	.005	.02
Sex (male), n (%)^h^		1780 (65.2)	1950 (66)	2230 (66.6)	.56	.28	.63	.55
Witnessed arrest, n (%)		1580 (58)	1860 (63.1)	2086 (62.9)	<.001	<.001	.92	<.001
**Arrest location, n (%)**								
	Home or residence	1625 (59.6)	1899 (64.4)	2066 (62.3)	<.001	.03	.09	.001
**Bystander response, n (%)**								
	Bystander CPR^i^	1443 (56.1)	1572 (54.5)	1798 (55.8)	.23	.80	.31	.43
	AED^j^ use by bystander	43 (1.7)	26 (0.9)	54 (1.7)	.01	.98	.008	.02
**First monitored rhythm, n (%)**								
	Shockable rhythm	496 (19.1)	518 (18.1)	538 (16.9)	.38	.03	.21	.10
Prehospital defibrillation, n (%)		621 (23.5)	665 (23)	726 (22.3)	.67	.27	.51	.54
Prehospital adrenaline use, n (%)		505 (19.6)	720 (25.1)	656 (20.3)	<.001	.52	<.001	<.001
**Prehospital airway, n (%)**								
	Supraglottic airway	1685 (61.9)	2158 (73.3)	2408 (72.6)	<.001	<.001	.56	<.001
	Endotracheal intubation	246 (9)	154 (5.2)	116 (3.5)	<.001	<.001	<.001	<.001
Cardiac arrest cognition to ED^k^ arrival (min), median (IQR)		33 (26-44)	35 (29-45)	36 (28-46)	<.001	<.001	.39	<.001
EMS^l^ call to EMS arrival (min), median (IQR)		7 (5-10)	9 (7-12)	9 (7-13)	.001	<.001	<.001	<.001
Arrest place arrival to departure (min), median (IQR)		13 (9-18)	15 (11-19)	15 (11-19)	<.001	<.001	.45	<.001
Arrest place departure to ED arrival (min), median (IQR)		9 (6-13)	10 (7-14)	10 (7-15)	<.001	<.001	.31	<.001
Cardiac arrest cognition to prehospital ROSC^m^ (min), median (IQR)		16 (11-25)	20 (12-28)	19 (13-26)	.003	.03	.29	.008

^a^Prepandemic period: from January to December 2019.

^b^Early phase pandemic period: from July 2020 to June 2021.

^c^Late phase pandemic period: from July 2021 to June 2022.

^d^Mann-Whitney U test (for continuous variables).

^e^*P*<.05 was significant.

^f^Kruskal-Wallis test (for continuous variables).

^g^Continuous variables are presented as median (IQR) and tested by using the Mann-Whitney *U* or Kruskal-Wallis test.

^h^Categorical variables are presented as n (%) and tested by using the chi-squared test.

^i^CPR: cardiopulmonary resuscitation.

^j^AED: automated external defibrillator.

^k^ED: emergency department.

^l^EMS: emergency medical services.

^m^ROSC: return of spontaneous circulation.

**Table 2:** Comparison of primary and secondary outcomes between prepandemic and pandemic periods using the nationwide, multicenter, out-of-hospital cardiac arrest registry of South Korea

Outcomes	Prepandemic period^a^ (n=2728)	Early phase pandemic period^b^ (n=2954)	Late phase pandemic period^c^ (n=3349)	*P* value (pre- vs early pandemic)	*P* value (pre vs late pandemic)	*P* value (early vs late pandemic)	*P* value
Survival to hospital admission, n (%)^d^	734 (26.9)	734 (24.8)	815 (24.3)	.08	.02	.64	.06
Survival to discharge, n (%)	385 (14.1)	355 (12)	392 (11.7)	.02	.005	.70	.01
Good neurologic outcome, n (%)	260 (9.5)	234 (7.9)	265 (7.9)	.03	.03	.99	.04

^a^Prepandemic period: from January 2019 to December 2019.

^b^Early phase pandemic period: from July 2020 to June 2021.

^c^Late phase pandemic period: from July 2021 to June 2022.

^d^Variables are presented as n (%) and tested by using the chi-squared test.

Multivariate analyses of the factors related to survival and neurological outcomes were performed to adjust for confounders that could affect the primary outcome (Table 3). Significant independent effects on all outcomes were observed for age (*P*<.001), witnessed arrest (*P*<.001), arrest at home or residence (*P*<.001), first monitored shockable rhythm (*P*<.001), prehospital adrenal use (*P*<.001 to *P*=.003), and supraglottic airway (*P*<.001). Cardiac arrest cognition at ED arrival significantly affected GNO after adjusting for confounders (adjusted odds ratio 1.001, 95% CI 1.000-1.002; *P*=.04). The difference in period (prepandemic, early phase, or late phase) did not independently affect any of the 3 outcomes.

**Table 3:** Multivariate analysis of factors affecting survival and neurological outcomes using the nationwide, multicenter, out-of-hospital cardiac arrest registry of South Korea.

Factors		Survival to hospital admission		Survival to discharge			Good neurologic outcome	
		Adjusted OR^a^ (95% CI)	*P* value	Adjusted OR (95% CI)	*P* value	Adjusted OR (95% CI)		*P* value
Age (per year)		0.971 (0.967-0.974)	<.001	0.965 (0.960-0.970)	<.001	0.961 (0.955-0.967)		<.001
Witnessed arrest (yes or no)		1.993 (1.747-2.275)	<.001	2.122 (1.732-2.600)	<.001	1.952 (1.517-2.513)		<.001
Arrest at home or residence (yes or no)		0.709 (0.628-0.799)	<.001	0.599 (0.507-0.708)	<.001	0.634 (0.519-0.776)		<.001
AED^b^ use by bystander (yes or no)		0.845 (0.513-1.396)	.51	1.530 (0.861-2.717)	.15	1.740 (0.918-3.298)		.09
First monitored rhythm (shockable or nonshockable)		4.667 (4.080-5.338)	<.001	9.061 (7.662-10.716)	<.001	13.945 (11.282-17.236)		<.001
Prehospital adrenaline (used or not used)		0.756 (0.651-0.877)	<.001	0.723 (0.583-0.896)	.003	0.553 (0.423-0.723)		<.001
Prehospital supraglottic airway (performed or not performed)		0.750 (0.654-0.861)	<.001	0.455 (0.380-0.545)	<.001	0.416 (0.336-0.514)		<.001
Prehospital endotracheal intubation (performed or not performed)		0.867 (0.657-1.145)	.32	0.556 (0.374-0.826)	.004	0.519 (0.319-0.844)		.008
Cardiac arrest cognition to ED^c^ arrival (per min)		1.000 (1.000-1.001)	.48	1.001 (1.000-1.002)	.052	1.001 (1.000-1.002)		.04
**Period when OHCA^d^ occurred**								
	Prepandemic period	Reference	—^e^	Reference	—	Reference		—
	Early phase pandemic period	0.961 (0.867-1.065)	.45	0.995 (0.861-1.150)	.95	0.993 (0.835-1.179)		.93
	Late phase pandemic period	1.015 (0.918-1.123)	.77	0.962 (0.836-1.107)	.59	1.026 (0.866-1.216)		.76

^a^OR: odds ratio.

^b^AED: automated external defibrillator.

^c^ED: emergency department.

^d^OHCA: out-of-hospital cardiac arrest.

^e^Not applicable.

## Discussion

### Principal Findings

In this study, we aimed to investigate changes in OHCA characteristics and outcomes during the SARS-CoV-2 pandemic using multicenter registry-based data. The incidence of OHCA increased, and neurologic and survival outcomes were worse during the pandemic era compared to the prepandemic era. Outcomes were particularly poor in the late phase when the number of SARS-CoV-2 infection cases spiked due to the spread of the Delta and Omicron variants [21].

During the pandemic, a significant increase in the global incidence of OHCA was observed compared to the prepandemic period. In cities such as New York, Detroit, and London, the number of OHCA cases increased proportionally with the number of COVID-19 cases during the initial surge [17-19]. In South Korea, the number of out-of-hospital sudden cardiac arrests increased by approximately 8%, from 60 per 100,000 population in 2019 (the year preceding the COVID-19) to 64.7 per 100,000 population in 2021 [22]. One meta-analysis that was reported early in the pandemic, which included 10 studies with a total of 35,000 participants published before October 2020, found that the incidence of OHCA increased by approximately 2.2 fold compared to the prepandemic period [10].

Globally, the outcomes of OHCA during the pandemic have consistently been reported to be less favorable than those observed before the pandemic [13]. For instance, in 2 regions of the United States, the survival-to-discharge rate decreased from 14.7% in 2019 to 7.9% in 2020 [23]. Meanwhile, in Singapore, while the incidence of OHCA increased during the pandemic, prehospital ROSC decreased [24]. In South Korea, the survival-to-discharge rate of OHCA, which had been steadily increasing for more than a decade before the pandemic, dropped from 8.7% in 2019 to 7.5% in 2020 and 7.3% in 2021. Moreover, the rate of brain function recovery at discharge reached 5.4% in 2019 but dropped to 4.9% in 2020 and 4.4% in 2021 [22]. In addition, a previous meta-analysis reported that the outcomes of OHCA were significantly poorer than before the pandemic in most of the included studies. The meta-analysis also showed a significant increase in mortality [10,25].

The characteristics of OHCA shifted during the pandemic periods in this study. Instances of witnessed OHCA and arrests at home or residence became more frequent in the pandemic era. This change has been observed in previous studies and can be attributed to the increased time spent at home during the pandemic due to quarantine and social distancing [10,26]. These results may also reflect the number of patients who progressed to OHCA due to being unable to visit a hospital in a timely manner as a result of reduced access to hospitals. Although bystander CPR decreased slightly during the pandemic, the difference was not statistically significant. This study also found that bystander use of AEDs decreased in the early phase of the pandemic but returned to prepandemic levels in the late phase of the pandemic. This is likely a reflection of bystander fears of COVID-19 transmission in the early phase of the pandemic [27]. A previous meta-analysis reported a decreased proportion of OHCAs with shockable rhythms during the pandemic [10,25]. In our research, the frequency of the first monitored shockable rhythm decreased progressively. The rise in respiratory arrests due to respiratory failure due to COVID-19 may have contributed to the diminished proportion of OHCA cases with shockable rhythms. Such cases would likely have a more unfavorable prognosis than OHCA cases of shockable rhythm with an etiology of cardiac origin [28]. Notably, the age demographic skewed older in the late phase of the pandemic, suggesting a possible increase in OHCA among older patients, who are more vulnerable to COVID-19.

Prehospital airway management has also been affected by the pandemic. An updated meta-analysis reported an increase in the use of both endotracheal intubation and supraglottic airway devices during the pandemic [25]. However, in this study, the supraglottic airway was favored, while endotracheal intubation progressively decreased during the pandemic. This is likely due to the EMS personnel’s fear of contracting COVID-19 during endotracheal intubation and the physical restrictions imposed by wearing protective clothing, face shields, and goggles. The summed rates of supraglottic airway and endotracheal intubation was higher than that before the pandemic, suggesting that more advanced resuscitation was performed in the prehospital setting.

Transport time, including the time from cardiac arrest diagnosis to ED arrival, significantly increased during the pandemic era. During the surge in patients with COVID-19, the lack of medical resources, poor access to hospitals, requirements for ambulance disinfection, and the need to wear protective equipment may have contributed to the increase in transportation time [29]. Difficulties in hospital selection led to prolonged, unnecessary prehospital treatment, and the frequency of adrenaline use increased during the pandemic. Further, prolonged transport time may have directly contributed to poorer outcomes in patients with OHCA during the pandemic. Furthermore, it emerged as an independent factor for poorer neurological outcomes in the multivariate analysis of this study. Such delays could compromise the continuity and quality of advanced life support from prehospital settings to hospitals, as underscored in the CoS, ultimately leading to suboptimal patient outcomes.

The prehospital factors consistently emphasized in the CoS, which play a key role in OHCA outcomes, were also heavily impacted by the pandemic. Maintaining a CoS in the event of future outbreaks and the emergence of new variants should be an ongoing discussion.

### Limitations

This study has several limitations. First, depending on the country and region, the impact of the COVID pandemic on OHCA may vary. Our findings were derived using only patients with OHCA in South Korea, and there may be limits to extending the results globally. Second, although the KoCARC registry was prospectively collected using established protocols and methods, the aim to examine the impact of the pandemic on OHCA was not predetermined before the initiation of the registry. Third, we were unable to investigate whether SARS-CoV-2 infection was the direct cause of cardiac arrest in the enrolled patients with OHCA. However, the main contribution of this study was to investigate the impact of prehospital factors associated with the CoS during the pandemic. Forth, despite the comprehensive data collection employing Utstein-style templates within the registry, the potential remains for latent confounders to influence the results. Lastly, long-term prognoses were not included in this analysis due to the limited observation period.

### Conclusions

The incidence of OHCA increased during the pandemic, while both the survival rate and GNO at discharge decreased, especially during community COVID-19 surges. Prehospital OHCA factors, which directly influence OHCA prognosis, were adversely affected by the pandemic. Ongoing discussions are needed to maintain the CoS in anticipation of future pandemics.

## Abbreviations

AED: automated external defibrillator
CoS: chain of survival
CPC: Cerebral Performance Category
CPR: cardiopulmonary resuscitation
ED: emergency department
EMS: emergency medical services
GNO: good neurologic outcome
KoCARC: Korean Cardiac Arrest Resuscitation Consortium
OHCA: out-of-hospital cardiac arrest
ROSC: return of spontaneous circulation

## Data Availability

The data sets used and analyzed during the current study are available from the corresponding author on reasonable request.
